# BiOCl/WS_2_ hybrid nanosheet (2D/2D) heterojunctions for visible-light-driven photocatalytic degradation of organic/inorganic water pollutants

**DOI:** 10.1039/d0ra02916e

**Published:** 2020-07-01

**Authors:** Waseem Ashraf, Shikha Bansal, Vikrant Singh, Sanmitra Barman, Manika Khanuja

**Affiliations:** Centre for Nanoscience and Nanotechnology, Jamia Millia Islamia New Delhi-110025 India manikakhanuja@gmail.com; Center for Advanced Materials and Devices, BML Munjal University Haryana-122413 India

## Abstract

This report presents the superior visible-light-driven photocatalytic response of novel 2D/2D BiOCl/WS_2_ (BW_*X*_) hybrid nanosheet heterojunctions prepared by a simple solution based sonochemical technique. These BW_*X*_ hybrid nanosheets are composed of 2D transition metal dichalcogenide material WS_2_ and BiOCl nanosheets. The comparative study of photocatalytic activity of BiOCl and BiOCl/WS_2_ hybrid nanosheets is carried out *via* photodegradation of Malachite Green (MG) and photoreduction of heavy metal ion Cr(vi) under visible light irradiation. The quantum efficiency of the samples is estimated in terms of the incident photon to electron conversion efficiency (IPCE) measurements. Nearly 98.4% of the MG degradation was achieved over BiOCl/WS_2_ (2%) photocatalyst in 45 min of irradiation. BiOCl/WS_2_ (2%) hybrid nanosheet catalyst showed the highest external quantum efficiency (EQE) in both the UV and visible regimes. This accomplishment demonstrated the promise of commercial application of the 2D/2D BiOCl/WS_2_ (2%) hybrid nanosheet photocatalyst.

## Abstract 2

Achieved highest photocatalytic performance of 2D/2D BiOCl/WS_2_ (2%) hybrid nanosheets for MG degradation (∼98.4% in 45 min) and Cr(vi) reduction.

## Graphical abstract

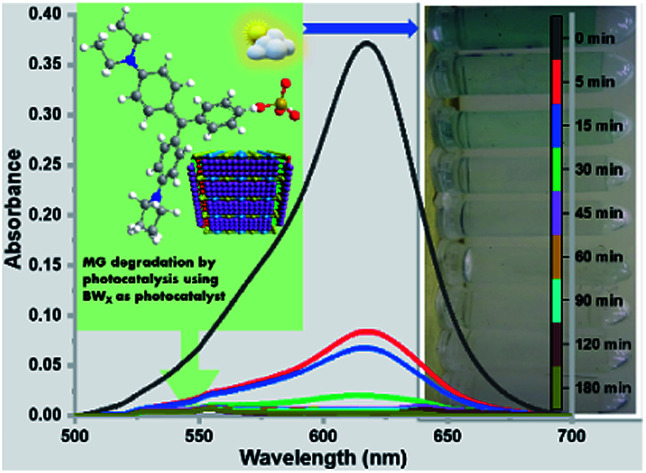

## 1. Introduction

The crises of global energy and increasing pollution in the environment are the two major concerns that the whole world would be looking for immediate yet sustainable solutions.^1,2^ The utilization of non-conventional sources of energy such as wind and visible light can be the most suitable remediation for the energy crisis and pollution. Among the sources of different renewable energy, using solar energy for the degradation of pollutants is of high demand.^3–5^ Since the first report on TiO_2_ catalyzed water splitting under the UV irradiation by Fujishima and Honda,^6^ there has been a plethora of examples of hydrogen gas production *via* water splitting using solar energy and water disinfection by using photocatalytic degradation of water pollutants.^7–14^ The photocatalytic degradation of water pollutants is a very promising technology being an eco-friendly, low cost technique and having a lack of any consequential contamination.^15–17^ A large variety of semiconductor nano-heterostructures have been proposed and fabricated to date that contribute to the advancement of photocatalytic conversion technology.^18–21^ However, making a photocatalyst with high efficiency, low recombination rate and maximum visible light absorption in the full spectral range is still a challenge.

The most commonly used organic dyes in the textile, pharmaceutical, and cosmetic industries are Methyl orange; Malachite green; Rhodamine B; Methylene blue; Congo red, *etc.* When these organic dyes are discharged into water resources, they can be very harmful.^22–25^ Malachite Green (MG), a water-soluble cationic dye used as an antifungal, antimicrobial and a topical antiseptic in aquaculture since 1930,^26,27^ has a structure similar to carcinogenic triphenylmethane dyes. Besides, the effluent containing Cr(vi) ions, the inorganic heavy metal water pollutant, is very toxic and detrimental to the environment.^28^ Various common techniques are being used to take out Cr(vi) from water *viz.* ultra-filtration, adsorption, reverse osmosis, *etc.*^29^ However, these methods have high power consumption and high operating cost. In an alternate approach for the removal of organic/inorganic pollutants of water, the development of an efficient photocatalyst that can work in the entire solar spectrum (that reaches our earth's surface), is of utmost priority because of low operating cost.^30^ Many materials, like α-Ga_2_O_3_, activated carbon from rice husk, and *Cocos nucifera* has been investigated and used as photocatalysts by researchers.^31^ However, the degradation efficiency of these materials was found to be less than 65% even after using a comparatively higher dosage of catalyst. This lead the researchers to find a new class of materials that include, Fe doped TiO_2_, Ag_3_PO_4_ modified by MWCNT's, UiO-66-NH_2_(Zr/Hf) and z-scheme based photocatalysts which increased the degradation efficiency to about 85%, but with high photocatalyst dosage. Also, the synthesis of this class of materials is complicated in comparison to the photocatalyst synthesized in the present work.^29,32^

In the last few years, bismuth-based semiconductors *viz.* bismuth oxyhalides have attracted many researchers due to their large optical and chemical stability, non-toxicity, cost-effectiveness, and potential application in photocatalytic energy conversion and water disinfection.^30,33^ One of the oxyhalides of bismuth, BiOCl, a ternary semiconductor (V–VII), has a wide bandgap ranging from ∼3.17–3.54 eV (ref. 34) possessing a layer-type structure consisting of [Bi_2_O_2_]^2+^ and Cl^−^ located in between the layers.^35^ This type of layered geometry facilitates the generation of an internal electric field useful for escalating the separation of the photo-induced electron–hole pairs leading to photoactivity.^36^ BiOCl generally gives higher photocatalytic response with UV-light irradiation in comparison to TiO_2_, due to its layer-type geometry.^37^ Besides, the wide bandgap of BiOCl degrades the photocatalytic activity in the visible-light regime. Hence, the extension of the photocatalytic properties of BiOCl nanostructures in the visible region has become a topic of interest for many researchers. Various methods, like dehalogenation, surface functionalization, photosensitization effect, metal/non-metal doping, and heterojunction constructions are being applied to modify BiOCl for the enhancement of absorption of light in the UV-visible regime.^38–43^ Among these, methods, making a heterojunction composed of BiOCl and another semiconductor with narrow bandgap lying in the visible regime having suitably positioned valence band maxima and conduction band minima, can be promising in the enhancement of visible-light harvesting, decreasing the electron–hole recombination rate and increasing the lifespan of the charge carriers. Numerous combinations of heterojunction systems, *e.g.* Bi_2_WO_6_/BiOCl, Bi_2_S_3_/BiOCl, g-C_3_N_4_/BiOCl, BiOCl/RGO, BiOI/BiOCl, CdS/BiOCl, WO_3_/BiOCl, BiVO_4_/BiOCl, NaBiO_3_/BiOCl, BiOCl/TiO_2_, *etc.*^44–49^ have been investigated by many researchers. It has been found that these heterojunctions show enhanced photocatalytic performance in comparison to their counterparts. However, there is only a single report present to date on the BiOCl/WS_2_ heterostructure system showing the enhanced photocatalytic response in degrading complex organic dyes.^49^

Tungsten disulfide (WS_2_) belongs to the family of 2D transition metal dichalcogenides (TMDs). It possesses a direct bandgap in the range ∼1.35–2 eV that leads to the absorption of light to 910 nm.^50^ So, the combination of BiOCl and WS_2_ nanosheets can significantly improve the spectrum absorption efficiency *via* bandgap modification and efficient charge transfer. Hence, a detailed investigation of the BiOCl/WS_2_ heterostructure system is important for the water remediation by photocatalytic degradation of the pollutants.

In the present study, the 2D/2D nanostructure composite of WS_2_ nanosheets and BiOCl nanosheets were prepared by using a simple solution based sonochemical technique. The study of photodegradation of Malachite Green (MG) and the photoreduction of a very toxic heavy metal ion Cr(vi)) was carried out under visible light irradiation using BiOCl/WS_2_ hybrid nanosheets. The photocatalytic response of BiOCl/WS_2_ was significantly affected by the amount of WS_2_ incorporated. The combination of BiOCl and WS_2_ nanosheets resulted in the enhancement of the charge carriers' transfer rate, resulting in better photocatalytic activity.

## 2. Experimental details

### 2.1 Reagents and materials

In the present work, all the chemicals used were of analytical grade unless otherwise mentioned and were used as they were bought from the supplier without any additional purification. Sodium tungstate dihydrate (Na_2_WO_4_·2H_2_O, 96%) and thiourea (CH_4_N_2_S) were bought from the Thermo Fischer Scientific India (P) Ltd., Mumbai, India. Hydroxylamine hydrochloride (NH_2_OH·HCl, 98%) and *N*-cetyl-*N*,*N*,*N*-trimethyl ammonium bromide (CTAB, 98%) were purchased from Central Drug House (P) Ltd., New Delhi, India. The pH value of the solution was maintained using buffer capsules (Merck, India). Potassium dichromate (K_2_Cr_2_O_7_, Merck, India, 99.99%) was used as a source of chromium. Bismuth nitrate pentahydrate (reagent grade, Bi (NO_3_)_3_·5H_2_O, 98%), HCl (37%), NaOH (≥97%, pellets), *n*-propanol (99.7%), and ethanol (99.9%, AR grade) were sourced from Sigma Aldrich, India.

### 2.2 Synthesis of WS_2_ nanosheets

The solution was prepared by mixing 0.005 M sodium tungstate, 0.02 M thiourea, and 0.01 M hydroxylamine hydrochloride in 30 ml DI water. In this solution, 0.24 g CTAB was added and stirred constantly for 60 min. The measured pH value of the white-colored precipitate thus formed was 6.15. The solution was then poured into a teflon lined autoclave. For 24 h the autoclave was kept in a furnace at a temperature of 180 °C. After 24 h the autoclave was taken out from the furnace and kept at room temperature for natural cooling. After natural cooling, the solution was filtered. The solid content thus recovered was washed with DI water and ethanol. Then the content was dried at a temperature of 30 °C inside the furnace for 4 h.^51^ The chemical reactions involved in the synthesis of WS_2_ nanosheets are as follows:

1 4CH_4_N_2_S + Na_2_WO_4_ + 4H_2_O → Na_2_WS_4_ + 8NH_3_ + 4CO_2_

2 Na_2_WS_4_ + 2NH_2_OH·HCl → WS_2_ + 2H_2_S + 2NaCl + N_2_+ 2H_2_O

### 2.3 Synthesis of WS_2_ nanosheets and BiOCl nanosheets hybrid

**Fig. 1:**
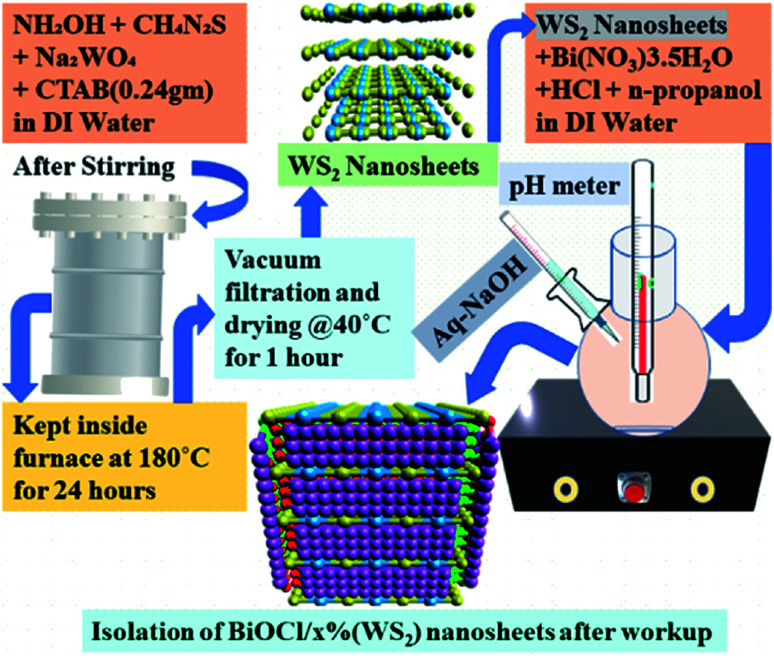
Schematic diagram of the synthesis of BW_*X*_ (*X* varies from 0% to 5%) hybrid nanosheets.

A solution of *n*-propanol (10 ml) and hydrochloric acid (2 M, 25 ml) was prepared in 20 ml DI water. In this solution, 1.86 g Bi(NO_3_)_3_·5H_2_O and a measured amount of WS_2_ nanosheets were added. The whole solution thus formed was then ultrasonicated for 30 min to get a uniform suspension. The suspension thus formed was turned white steadily after adding ∼10 ml of 4 M NaOH aqueous solution, drop by drop. The pH value of the mixture was found to be 8.0. The suspension was mixed with stirrer for 30 min and then kept as it is for 6 h. The formed resultant precipitates formed were centrifuged and washed using ethanol and DI water thoroughly. At last, the precipitates were dried at 100 °C for 12 h to achieve BiOCl/WS_2_ hybrid nanosheets (Fig. 1).

A series of BiOCl/WS_2_ hybrid nanosheets by varying the wt% of WS_2_ from 0% to 5% were synthesized and denoted as BW_*X*_ hybrid nanosheets, where *X* denotes the weight percentage of WS_2_ nanosheets to BiOCl. Pure BiOCl nanosheets (BW_0_) were synthesized under similar conditions without the addition of WS_2_ nanosheets.

### 2.4 Characterization

FESEM (Field Emission Scanning Electron Microscopy) study was carried out using Zeiss, Sigma Field Emission Scanning Electron Microscope, to investigate the morphology of the samples. EDX (Energy Dispersive X-ray) spectra taken from FESEM were used for composition analysis. Crystal structure and phase identification studies were carried out through X-ray diffractogram (XRD Smart Lab Guidance, Rigaku) were recorded by using Cu K_α_ source with wavelength, *λ* = 1.5418 Å. The Raman scattering measurements were conducted using Alpha 300 RAS TS-150 Witec Confocal Raman Microprobe equipped with 365 nm and 532 nm argon-ion lasers. The absorption of light in 300–800 nm range was investigated by UV-vis diffuse reflectance spectroscopy (UV-vis Cary Series, Agilent Technologies). Photocatalytic activity of the BW_*X*_ hybrid nanosheets for MG degradation and highly toxic Cr(vi) ion reduction was analyzed using the UV-vis spectrometer (UV-vis Cary Series, Agilent Technologies). Fourier transform infrared spectroscopy (FTIR) (Vertex 70V, Bruker) was used to study the functional groups and bond structure. The lifetime of charge carriers was evaluated by using the Time-Correlated Single-Photon Counting (TCSPC) (Horiba DeltaFlex-01-DD) measurement spectrometer at an excitonic emission wavelength of 280 nm with typical short optical pulses from <100 ps optical FWHM, spectral FWHM < 15 nm in pulsed mode, 100 MHz maximum repetition rate, and with an instrument response function of ∼200 ps. The emission decays were deconvolved with IRF using EZ time software. The double-exponential kinetics model was used to fit the decay curves of the samples and the fitting was done in the best fitness limits, with *χ*^2^ in the range 1–1.2. The relationship between the photoactivity improvement and the wavelength of the incident light, and the external quantum efficiency of the samples were investigated by using Incident Photon to Converted Electron (IPCE) studies. For IPCE measurements, 100 mg of photocatalyst was compressed under a pelletizer using a 10 mm stainless steel die and the applied force was 50 kN. The thin pellet with 10 mm diameter was annealed for 2 hours at a temperature of 50 °C. For electrode preparation, silver paste was applied to one of the faces of the pellet and a thin copper wire was placed on the silver pasted face and again the silver paste was applied to the copper wire at the contact point as well to make the ohmic contact. The silver paste was allowed to dry for at least 1 hour to make sure that the proper contact is established. The other end of the wire was inserted through thin hollow glass pipe with 2 mm diameter and 3-inch height. One end of the pipe was kept close to the edge of the pellet and at this end, resin was applied to the copper wire, silver pasted face, edge of pellet and thin pipe in such a way that only the bare side of pellet could come in contact with electrolyte when dipped in it. In this case, 1 molar sodium sulphate (Na_2_SO_4_) was used as an electrolyte. After the whole setup was kept in place, a linear sweep voltammetry was performed with the help of Autolab PGSTAT302N potentiostat and simultaneously the bare face of the sample was illuminated with the help of monochromator which was controlled by lab view programme to automatically select a particular wavelength (in the range of 200–700 nm) for measurement of electrode current as a function of wavelength.

### 2.5 Photocatalytic experiment

As-synthesized samples were investigated for their photocatalytic properties by Malachite Green (MG) degradation under visible light. A green-colored solution was prepared by mixing 1 mg of MG in 100 ml of DI water. The solution was then kept in the dark for half an hour. After half an hour, 50 mg BW_*X*_ (*X* = 0%, 2%, and 4%) was added in the solution. Again, the solution was kept in the dark for half an hour. Then, the solution was irradiated in visible light by using xenon arc lamp with a power of 100 mW cm^−2^ with AM 1.5 filter. Immediately 1 ml solution was transferred into an eppendorf as the first sample and the subsequent samples were collected each after 15 min for 1.5 h. The collected samples were centrifuged to study the absorption spectra of supernatants. The photocatalytic performance of BW_*X*_ hybrid nanosheets was further tested for the Cr(vi) ions photoreduction. A solution of 1 mg of Cr(vi) ions was prepared in 100 ml of DI water. Then 50 mg of the photocatalyst was added in this solution. Taking the first sample at 0 min, 1 ml sample was taken out of the solution after every 15 min for 3 h. After this, the collected samples were kept for centrifugation and studied for the absorption spectroscopy. The photodegradation efficiency was calculated by using the formula:3
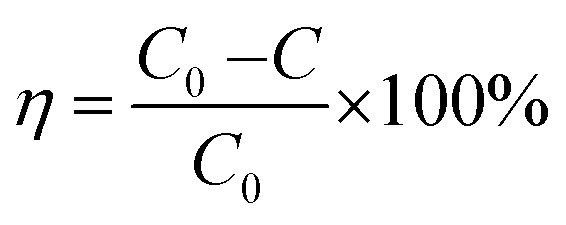
where *C*_0_ is the initial concentrations of MG/Cr(vi) solutions and *C* is the concentration at time *t*, after irradiation.

## 3. Results and discussion

### 3.1 Morphology and crystal structure

**Fig. 2:**
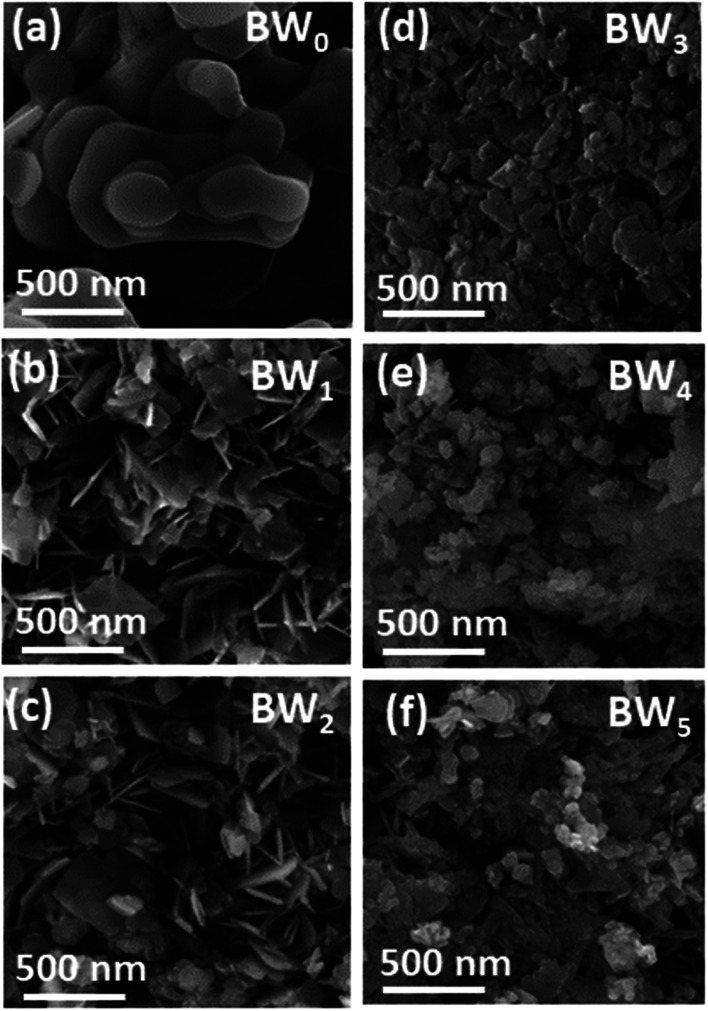
FESEM images of BW_*X*_ hybrid nanosheets where *X* varies from 0% to 5% prepared by sonochemical method.

All the samples' microstructure was studied by FESEM. Morphology in Fig. 2(a) of BW_0_ revealed a quite smooth surface with a nanosheet-like structure having round edges and different diameters. The hierarchical plate-like structure for BW_0_ was in agreement with the previous report.^52^Fig. 2(b and f) revealed that the WS_2_ incorporation significantly altered the morphology of BiOCl nanosheets in BW_*X*_ nanostructures. After the addition of WS_2_, the morphology of the heterojunctions changed to a sheet-like structure with sharp edges. However, the further increase in WS_2_ content (>3%) in BiOCl nanosheets resulted in the agglomeration of the nanosheets. This agglomeration leads to a decrease in active sites for light-harvesting and molecule adsorption.

**Fig. 3:**
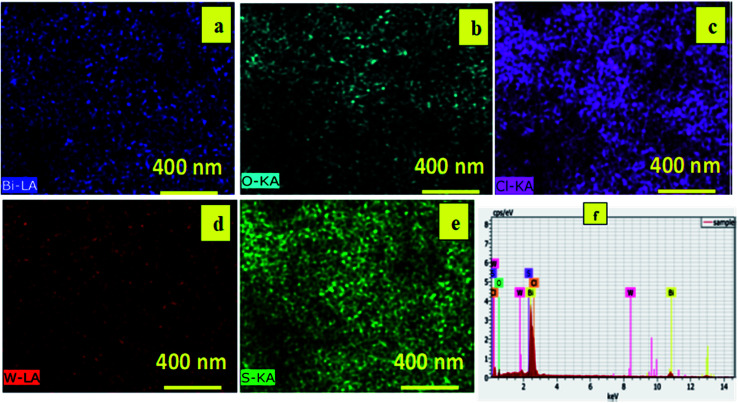
Elemental mapping of Bi, O, Cl, W and S on the BW_2_ nanosheets showing (a) blue, (b) aqua, (c) magenta (d) red (e) green, corresponding to bismuth, oxygen, chlorine, tungsten, and sulphur, respectively and (f) EDX spectra of BW_2_ nanosheets.

Energy dispersive X-ray (EDX) was used to study the elemental mapping of BW_2_ to confirm the existence of WS_2_ in the sample. Fig. 3 depicted that the W and S atoms are evenly spread on the surface of BW_2_ hybrid nanosheets, that confirmed the existence of WS_2_. The compositional mapping of BW_2_ is given in Table 1, quantitatively.

**Table 1:** Composition analysis of BW_2_ nanosheets

Element	Atomic concentration (%)	Weight concentration (%)
Bi	34	96
O	40	9
Cl	25	12
W	0.6	1.6
S	1.3	0.6

**Fig. 4:**
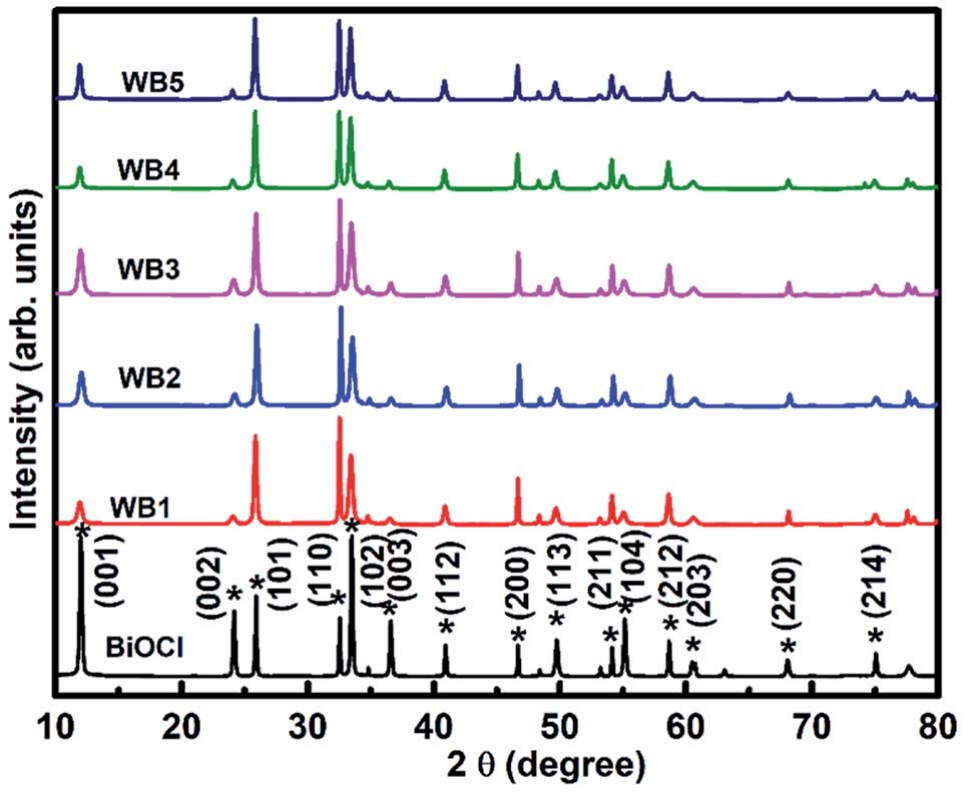
X-ray diffractograms of BW_*X*_ hybrid nanosheets where *X* varies from 0% to 5% prepared by the sonochemical method.

XRD patterns (Fig. 4) of BW_*X*_ (*X* varies from 0% to 5%) hybrid nanosheets were analyzed to study the crystallographic structure. The 2*θ* values corresponding to the observed peaks in all the samples were matched with the 2*θ* values of standard data (JCPDS # 73-2060) of the XRD peaks of pure BiOCl. It was found that the as-synthesized samples were pure in-phase and crystallized in the expected tetragonal matlockite structure. In all the samples, the XRD peaks displayed the obvious broadening. This broadening of the peaks was attributed to the nanoscale structure of the samples.

The BW_0_ nanosheets displayed a pronounced (001) texture. With the addition of WS_2_ in BiOCl nanosheets, crystal structure got reoriented from (001) texture to (110) texture followed by (101) texture. The intensity ratios of (110) and (001) peaks for all BW_*X*_ hybrid nanosheets are given in Table 2. This reorientation depicts the inhibition of crystal growth along the basal ‘*ab*’ plane in the case of BW_*X*_ hybrid nanosheets. As per literature, this texture/faceted helps in setting up internal electric fields, that support the electron–hole separation in this type of material.^53^ It was thus clearly indicated that increasing WS_2_ content was accountable for the crystal reorientation in these hybrid nanosheets. This kind of crystallographic reorientation was also being reported by Jia *et al.* in Bi_2_S_3_/BiOCl composites.^54^ The observed change in crystallographic orientation can be attributed to the transition from reduced surface termination to oxidized surface termination and the surface energy stability associated.

**Table 2:** Variation of lattice parameters, cell volume (*V*), and peak intensity ratio (*I*_110_/*I*_001_) of BW_*X*_ hybrid nanosheets where *X* varies from 0% to 5% with the change in WS_2_ content

Samples	*a* (Å)	*c* (Å)	*V* (Å^3^)	*D*_*hkl*_ (nm)	Intensity ratio (*I*_110_/*I*_001_)
BW_0_	3.888	7.358	111.228	118.49	0.41
BW_1_	3.888	7.413	112.059	105.41	5.03
BW_2_	3.881	7.336	110.496	102.65	3.05
BW_3_	3.887	7.374	111.412	98.1	2.13
BW_4_	3.882	7.412	111.698	81.49	3.72
BW_5_	3.881	7.416	111.701	79.71	2.32

The crystallite size of the hybrid nanosheets was evaluated from the Scherrer's formula^41^ given as follows:4
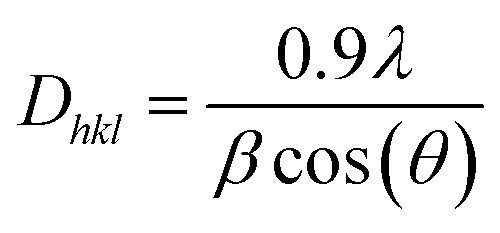
where *D*_*hkl*_ (nm) represents the average crystallite size, *λ* = 0.1540 nm, is the Cu K_α_ radiation's wavelength, *β* (radians) represents the full-width at half-maximum (FWHM) of the peak, and *θ* is the Bragg's diffraction angle.

It was discovered that the crystallite size decreases from 118.5 to 79.7 nm with the increase in WS_2_ content in the BiOCl matrix from 0% to 5% (Table 2). This indicates that the BW_*X*_ hybrid nanosheets possessed smaller crystallite sizes with a large surface area. In a tetragonal unit cell, the relationship between interplanar spacing ‘*d*’ of (*hkl*) planes and the lattice constants ‘*a*’ and ‘*c*’ is given as:5
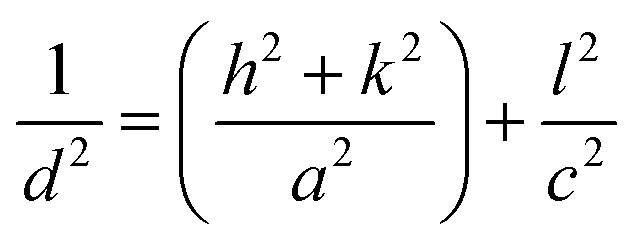


(001) and (200) planes were used to calculate the lattice constants all the samples. The calculated lattice parameters were found to agree with the standard values *a* = 3.887 Å, *c* = 7.354 Å, and *V*_0_ = 111.11 Å^3^ corresponding to BiOCl. The non-presence of XRD peaks corresponding to WS_2_ in the X-ray diffractogram of BW_*X*_ is attributed to the presence of WS_2_ in a very low amount in the BiOCl precursor. Similar results were reported in the case of CQDs/BiOI and Co_3_O_4_/BiOCl hybrids.^55,56^

**Fig. 5:**
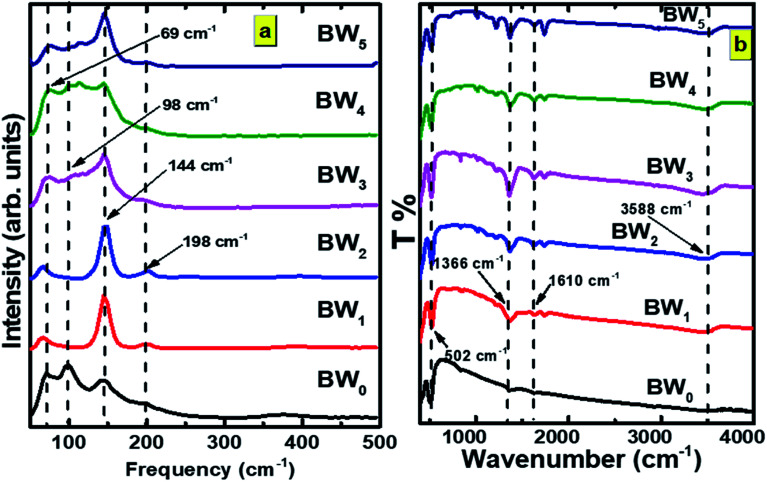
(a) Raman spectra and (b) FTIR spectra of BW_*X*_ hybrid nanosheets where *X* varies from 0% to 5% prepared by the sonochemical method.

Information regarding vibration along with the crystal structure and stress state was obtained from the Raman spectra of BW_*X*_ hybrid nanosheets (Fig. 5(a)).

Five Raman vibration bands were observed for BW_0_ at 69, 98, 144, 198, and 365 cm^−1^. Peaks at 144 and 198 cm^−1^ were dedicated to the A_1g_ and E_g_ internal mode of Bi–Cl stretching.^37^ Peaks at the positions 69 and 98 cm^−1^ were dedicated to the first-order scattering corresponding to E_g_ and A_1g_ modes of Bi.^57^ This depicts the presence of the unreacted Bi in the BiOCl matrix. It was observed that the BW_1_ and BW_2_ had a sharp peak at 142 cm^−1^ along with smaller intensity peaks at 67 and 196 cm^−1^. The high-intensity blue-shifted peak at 142 cm^−1^ followed by the intensity reduction of the other peaks indicated that the addition of WS_2_ nanosheets significantly affected the Bi–Cl internal A_1g_ stretching mode. The reduced degrees of freedom along ‘*a*’ and ‘*b*’ axes resulted in restricted stretching in the tetragonal crystal lattice of BiOCl.^58^ The blue shift of the Raman vibrational peaks in the BW_1_ and BW_2_ samples evidenced a strong coupling between the BiOCl and WS_2_ nanosheets. However, the nature of the Raman spectra for BW_3_, BW_4_, and BW_5_ quite similar to that of BW_0_.

FTIR measurements were carried out to further study the phase transformation of BiOCl and interfacial relations between BiOCl and WS_2_. FTIR spectra of BW_*X*_ nanostructures are presented in Fig. 5(b). Bi–O stretching vibration at around 502 cm^−1^ was observed in BW_0_ which is common in a tetragonal phase BiOCl crystal. O–H stretching frequency was found at 1366 cm^−1^ and 1610 cm^−1^ in BW_0_. A wide hump around 3588 cm^−1^ was dedicated to the presence of H_2_O in BiOCl.^32^ In the case of the BW_*X*_ hybrid nanosheets, the intensity of O–H stretching vibrations at 1366 cm^−1^ and 1610 cm^−1^ was increased significantly. This implies the presence of a large number of O–H groups at the surface of BW_*X*_ hybrid nanosheets. The stretching vibration observed around 671 cm^−1^ and 820–980 cm^−1^ could be allocated to W–S and S–S for the BW_*X*_ hybrid nanosheets.^42^ A steady intensification and a shift of the band slightly at 820–980 cm^−1^ in BW_*X*_ was due to the strong coupling of BiOCl and WS_2_ nanosheets. This strong coupling is vital to escalate the separation and transfer of the charge carriers and consequently increase the photocatalytic performance of the hybrid nanosheets. The prominent absorption band of W–S was not observed for the hybrid nanosheets, indicating that 2D WS_2_ nanosheets are well distributed over the BiOCl matrix.

### 3.2 Optical properties

**Fig. 6:**
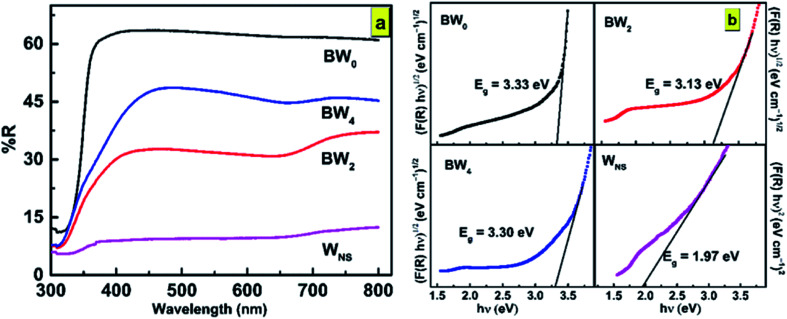
(a) UV-vis DRS spectra and (b) the plot of (*F*(*R*)*hν*)^1/2^*vs. hν* for determination of bandgap energies of BW_0_, BW_2_, BW_4_, and W_NS_ hybrid nanosheets.

From the photocatalysis point of view, one more significant property of BW_*X*_ is the absorption of light. The optical properties of BW_*X*_, (*X* = 0%, 2%, and 4%), hybrid nanosheets were evaluated by using the UV-vis diffuse reflectance spectroscopy (DRS). The DRS of the pure BiOCl nanosheets is different from the DRS of BW_*X*_ hybrid nanosheets (Fig. 6(a)). The BW_0_ nanosheets showed a sharp fundamental absorption edge at ∼372 nm due to intrinsic bandgap absorption. However, after the incorporation of WS_2_ nanosheets into BiOCl nanosheets, the absorption edge of the BW_*X*_ hybrid nanosheets was shifted to the lower wavelength. This difference leads to the change in the bandgap of these samples.

#### 3.2.1 Band gap calculations

The bandgap calculation for BW_0_, BW_2_, and BW_4_ hybrid nanosheets and W_NS_ was done by using Kubelka–Munk (K–M) theory from the respective diffuse reflectance spectra (DRS).^59,60^ The relation between the diffuse reflectance (*R*) and the absorbance of the material is given by Kubelka–Munk (K–M) function, *F*(*R*), as follows:6
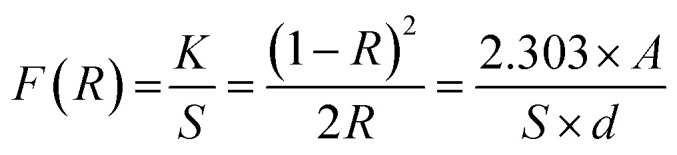
where *A* is the absorbance of the sample, *K* is the absorption coefficient, *S* is the scattering coefficient, and ‘*d*’ is the thickness of the sample. The scattering coefficient, ‘*S*’ accounts for the internal scattering. It depends upon the properties of the material like refractive index and particle size. If we take ‘*S*’ as a constant with respect to wavelength, then *F*(*R*) ∝ *α*, where ‘*α*’ represents the coefficient of absorption, written as:7
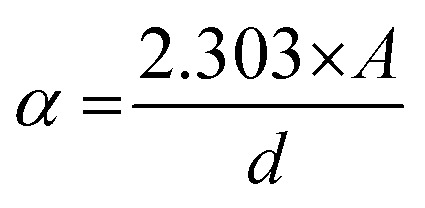


The bandgap energy (*E*_g_) is given by:8
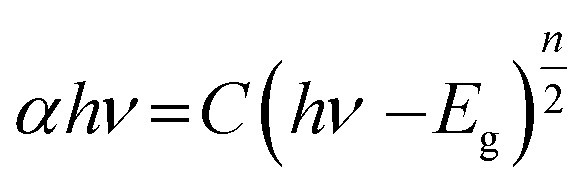
where ‘*h*’ represents the Plank's constant, ‘*ν*’ represents the frequency of light, ‘*C*’ represents the constant of proportionality, and ‘*E*_g_’ represents the bandgap energy. ‘*n*’ is an integer and its value is based on nature of the transition occur in a semiconductor material. *n* is taken as 4 for an indirect bandgap material and *n* is taken to be 1 for a direct bandgap material. BiOCl has an indirect bandgap. The value of *n* was taken to be 4 for BiOCl. However, *n* = 1 for WS_2_ as it possesses a direct bandgap transition.

The determination of the bandgap of BW_*X*_ (*X* varies from 0% to 5%) was done by using the graph: (*F*(*R*)*hν*)^1/2^*vs. hν* (Fig. 6(b)). The bandgap energy of the sample is obtained by extrapolating the linear portion of the corresponding (*F*(*R*)*hν*)^1/2^*vs. hν* plots at (*F*(*R*)*hν*)^1/2^ = 0. The bandgap energy of the BW_*X*_ samples is decreased from 3.33 eV to 3.13 eV when the amount of WS_2_ is increased from 0% to 4%. The strong coupling between BiOCl and WS_2_ nanosheets resulted in a significant bandgap narrowing near the interface. Hence, the sensitivity of the heterostructures towards the visible light is increased which significantly contributes to the visible-light-driven photocatalysis.

The position of *E*_VB_, the valence band edge potential and *E*_CB_, the conduction band edge potential for BW_*X*_ hybrid nanosheets, was calculated by using the Butler and Ginley equation.^59^

9 *E*_VB_ = *X* − *E*_e_ + 0.5*E*_g_

10 *E*_CB_ = *E*_VB_ − *E*_g_

‘*X*’ represents the absolute electronegativity of the semiconductor calculated from the electronegativity of the atoms, it is composed of, ‘*E*_e_’ (∼4.5 eV) represents the free electrons' energy on the hydrogen scale, and ‘*E*_g_’ represents the bandgap energy. The calculated *E*_VB_ and *E*_CB_ values corresponding to BW_0_, BW_2_, BW_4,_ and WS_2_ nanosheets are presented in Table 3.

**Table 3:** Calculated values of the energy band structure parameters of BW_0_, BW_2_, BW_4_ hybrid nanosheets, and WS_2_ nanosheets

Sample	*X* (eV)	*E*_g_ (eV)	*E*_VB_ (eV) *vs.* NHE	*E*_CB_ (eV) *vs.* NHE
BW_0_	6.36	3.33	3.525	0.195
BW_2_	6.36	3.13	3.425	0.295
BW_4_	6.36	3.30	3.01	−0.29
W_NS_	5.66	1.99	2.155	0.165

**Fig. 7:**
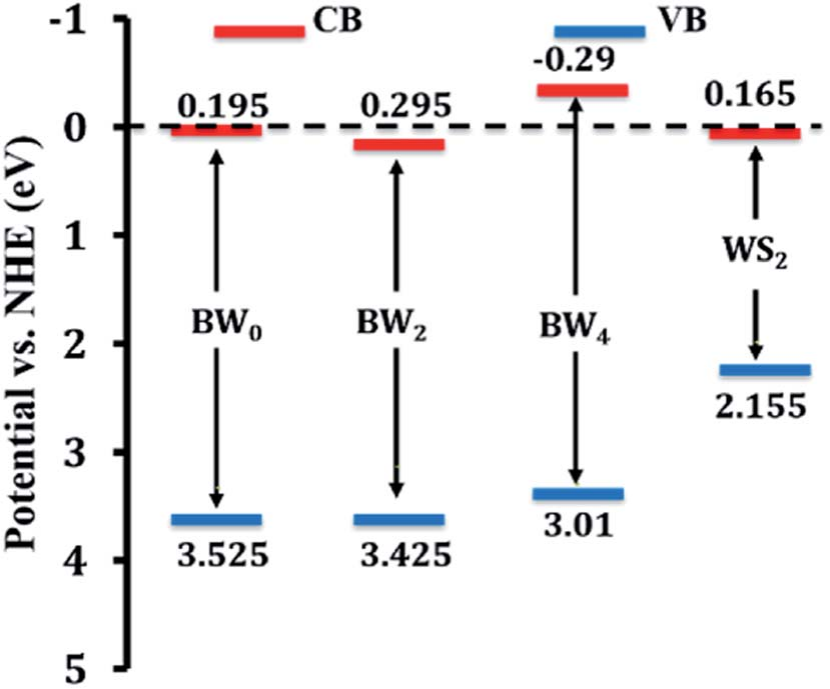
Schematic illustration of band edge positions of BW_0_, BW_2_, BW_4_ hybrid nanosheets and WS_2_ nanosheets.

The schematic arrangement of the estimated band edge positions of these samples is presented in Fig. 7.

### 3.3 Time-correlated single photon counting

**Fig. 8:**
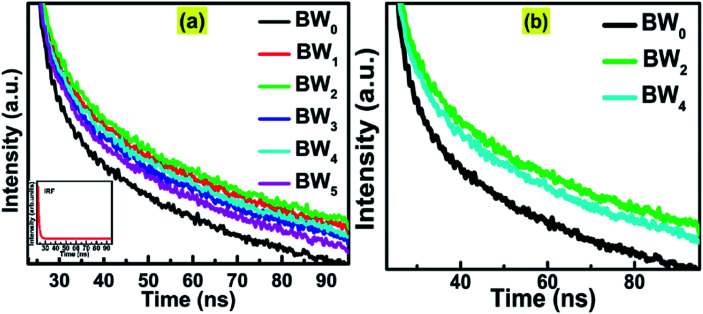
(a) Time-correlated single-photon counting (TCSPC) spectra of BW_*X*_ hybrid nanosheets where *X* varies from 0% to 5%. Inset shows the IRF data. (b) Magnified TCSPC spectra for BW_0_, BW_2_ and BW_4_ nanosheets.

The average lifetime of the excitons and dynamics of the photo-induced charge carriers was evaluated by Time-Correlated Single Photon Counting (TCSPC) study (Fig. 8).

The decay curves of the hybrids, BW_*X*_ (*X* varies from 0% to 5%), were simulated by using the double-exponential model.^61^11
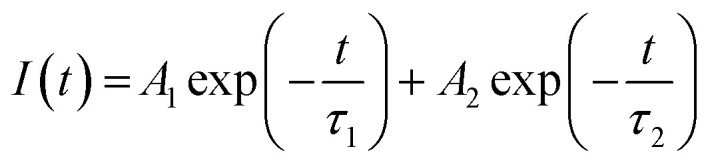
where *I*(*t*) represents the intensity of photoluminescence, *τ*_1_ and *τ*_2_ represent the decay times corresponding to slower and faster decay processes, respectively, and *A*_1_ and *A*_2_ represent the amplitudes. The inter-band exciton recombination results in a fast PL decay process, however, the slow PL decay process is based on the electron–hole pairs recombine in an indirect mode.^62^

For pure BiOCl nanosheets, the contribution of the fast decay process with a 6.5 ns lifetime (*τ*_2_), is 91.79% and slow decay process of 277.38 ns (*τ*_1_), contributes only 8.21%. This implies that almost 92% of the excited states are getting deactivated *via* non-radiative path. The incorporation of WS_2_ in BiOCl has a significant effect on the dynamics of the charge carriers. 1 wt% addition of WS_2_ slows down the decay process by increasing the contribution of *τ*_1_ from 8.2% to 22.36% along with the increase in both *τ*_1_ and *τ*_2_ at the cost of the contribution of *τ*_2_. As per Table 4, when the WS_2_ content was increased from 1 wt% to 2 wt%, there was a further increase in the contribution of *τ*_1_ from 22% to 26% and the fast, and the slow decay components were lengthened from 8.21 ns to 9.64 ns and 325.49 ns to 340.11 ns. This increment suggests the evolution of new radiative pathways which promote the transfer of a greater number of photoexcited electrons to BiOCl. Similar observations were reported in case of CdS–CdSnO_3_ composite.^20^ Further addition of WS_2_ (3 wt%) however shortened the time constant of both the fast and the slow decay components of BiOCl (6.95 ns and 285.41 ns, respectively). This depicted the trapping of charge carrier from WS_2_ with the addition of WS_2_ content beyond 3 wt% in BiOCl nanosheets. The excess of the charge carrier trapping boosts the electron–hole pair recombination in the nanostructures.^53^ At relatively higher WS_2_ content of 4 wt% and 5 wt%, electrons trapped at BiOCl were such abundant that it helped in the enhancement of recombination rate of the charge carriers across the BiOCl/WS_2_ interface. This outcome depicted that the content of WS_2_ beyond a certain optimum value might act as a booster for interfacial electron–hole pairing and the prolonged decay lifetime observed for BW_2_ demonstrated the improved charge transfer from WS_2_ to BiOCl.

**Table 4:** Simulated parameters of eqn (11) and the calculated average lifetime of the BW_*X*_ hybrid nanosheets (*X* varies from 0% to 5%)

Sample	Lifetime (ns)		Relative intensities (%)		Chi-squared
	*τ*_1_	*τ*_2_	*f*_1_	*f*_2_	*χ* ^2^
BW_0_	277.3	6.5	8.2%	91.8%	1.167
BW_1_	325.5	8.9	22.4%	78.6%	1.152
BW_2_	340.1	9.6	26.1%	73.9%	1.21
BW_3_	285.4	7.0	11.6%	88.4%	1.144
BW_4_	326.2	9.3	24.2%	75.8%	1.130
BW_5_	328.4	9.5	23.9%	76.1%	1.236

### 3.4 Photocatalytic response

**Fig. 9:**
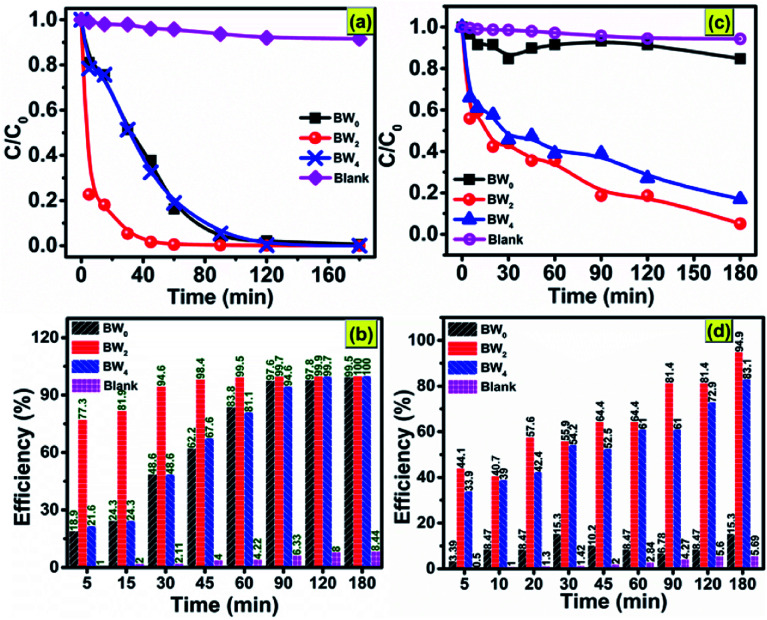
Photocatalytic degradation of (a) MG dye; (c) Cr(vi) without catalyst and in the presence of BW_0_, BW_2_, and BW_4_ under visible light irradiation; photocatalytic efficiency *vs.* time plot for (b) MG dye and (d) Cr(vi), over BW_0_, BW_2,_ and BW_4_ hybrid nanosheets.

The as-synthesized BW_0_, BW_2_, and BW_4_ hybrid nanosheets were analyzed for their photocatalytic response by Malachite Green (MG) degradation and Cr(vi) ion photoreduction under visible light irradiation. Fig. 9(a) shows the photodegradation of Malachite Green (MG) over BW_0_, BW_2_, and BW_4_ hybrid nanosheets. As can be seen in Fig. 9(b), after 30 min irradiation in visible light, ∼94.6% of MG was degraded over BW_2_ catalysts, whereas only 48.6% of MG was decomposed over BW_0_ and BW_4_ at the same time. Among all the BW_*X*_ (*X* varies from 0% to 5%) samples, BW_2_ showed the highest photocatalytic response towards MG degradation. This was attributed to the incorporated WS_2_ nanosheets that helped in structural reorientation and bandgap narrowing. The structure reorientation facilitated the development of internal electric fields that helped in the separation of charge carriers and were beneficial for the charge transfer (revealed by X-ray diffractograms and TCSPC spectra). And bandgap narrowing contributed towards the visible-light-harvesting (confirmed by DRS).

However, too much WS_2_ incorporation would result in decreasing the light absorption due to a lowering of the active sites as a result of agglomeration confirmed *via* FESEM images. Hence photocatalytic response got degraded upon increase the WS_2_ content beyond 2%. The degradation efficiency for MG reached ∼99.5% in 60 min, indicating the high photocatalytic response of BW_2_. The photocatalytic reduction of CrO_3_ solution using BW_0_, BW_2_, and BW_4_ hybrid nanosheets is shown in Fig. 9(c). It is demonstrated that the BW_2_ enabled the photoreduction of Cr(vi) up to ∼95% within 3 h of irradiation (Fig. 9(d)).

To date, only a few reports are present on the photocatalytic degradation of MG and Cr(vi) ions photocatalytic reduction using bismuth oxychloride-based catalysts under visible light irradiation. Some of these previous reports are mentioned in Table 5. Table 5 shows that the BiOCl/WS_2_ hybrid nanosheets prepared in the present work are better in the photocatalytic response than the existing bismuth oxychloride-based catalysts. The papers reporting more than 90% degradation efficiency have used a higher photocatalyst dose for a comparatively lower amount of dye to be degraded. The irradiation time taken for the photodegradation is also much higher than the irradiation time taken in the present work. Degradation efficiency ∼98.5% in only 45 min and more than 80% in 90 min was achieved in case of 10 mg l^−1^ of MG and Cr(vi) ions respectively, over only 0.5 g l^−1^ of BW_*X*_ hybrid nanosheets catalysts dosage. This result indicates that the 2D/2D BW_*X*_ hybrid nanosheet can be applied as an efficient photocatalyst for the degradation of complex and toxic organic dyes and heavy metal ion pollutants that are extremely hard to degrade.

**Table 5:** Previous reports on photocatalytic degradation of MG and Cr(vi) using BiOCl based materials

Catalyst	Dye conc. (mg l^−1^)	Dose (g l^−1^)	Irradiation time (min)	Dyes	Efficiency	Reference
BiOCl	25	0.7	120	MG	65%	63
Sn:BiOCl	10	—	240	MG	98%	64
Mn:BiOCl	25	0.7	480	MG	97%	40
Bi (Bi_2_S_3_)/BiOCl	40	20	40	MG	97.4%	65
BiOCl_0.5_Br_0.5_	10	—	100	MG	95%	66
BiOCl/Fe_2_O_3_	10	10	300	Cr(vi)	50%	67
BiOCl	30	—	12	Cr(vi)	100%	68
g-BN/BiOCl	10	0.8	150	Cr(vi)	90%	69
Ag/BiOCl	—	—	180	Cr(vi)	—	70
BiOCl/WS_2_	10	0.5	45	MG	98.4%	Present work
	10	0.5	120	Cr(vi)	94.9%	

In general, the experimental data of photocatalytic degradation is simulated by using the pseudo-first and second-order kinetic equations stated as follows:^71^

Pseudo-first-order kinetic equation, 12
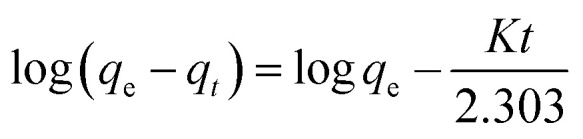
Pseudo-second-order kinetic equation, 13
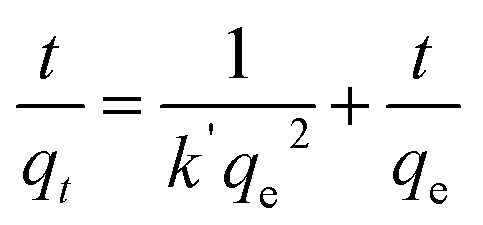
where *q*_e_ (mg g^−1^) represents the amounts of BW_*X*_ adsorbed at equilibrium and *q*_*t*_ (mg g^−1^) represents the amounts of BW_*X*_ adsorbed at time *t*. *K* (min^−1^) and *k*′ (g (mg^−1^ min^−1^)) represent the rate constants corresponding to pseudo-first and second-order adsorption, respectively.

**Fig. 10:**
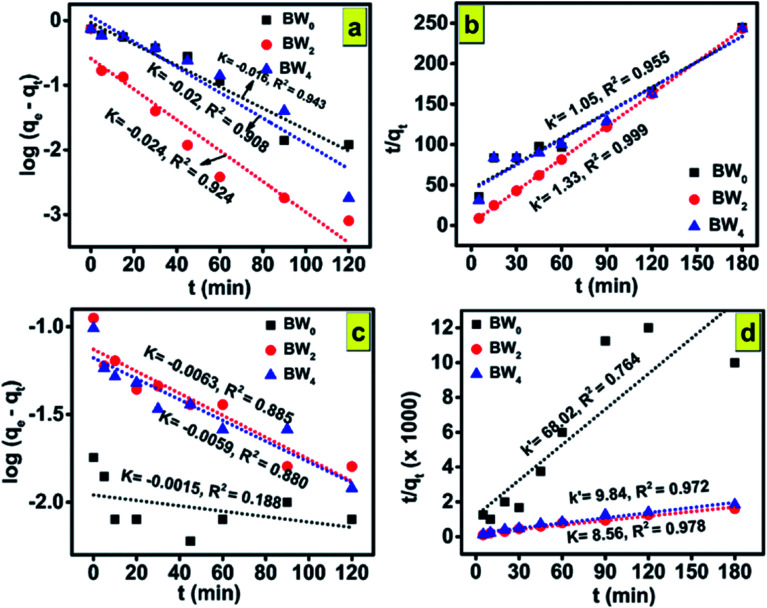
The adsorption mechanism model of (a and b) organic dye MG and (c and d) heavy metal ion Cr(vi) over BW_0_, BW_2_, and BW_4_ hybrid nanosheets. (a and c) Corresponds to the pseudo-first-order kinetics model; (b and d) corresponds to the pseudo-second-order kinetics model.

Fig. 10(a,c) and (b,d) and show the plots of experimental data for MG and Cr(vi), based on eqn (12) and (13), respectively. Table 6 (represents the absorption parameters as per eqn (12) and (13)) shows that the regression coefficient values (*R*^2^) of eqn (12) and (13) are greater than 0.90. Hence, both the models are being followed in case of adsorption of MG over BW_*X*_ hybrid nanosheets. Besides, in the case of Cr(vi) ions photoreduction, the *R*^2^ values (>0.90) of eqn (13) are greater than the *R*^2^ values (<0.90) of eqn (12). Hence, we can infer that the adsorption of Cr(vi) ions over BW_*X*_ hybrid nanosheets follows only the second-order equation.

**Table 6:** Parameters of eqn (12) and (13) determined by the adsorption of MG and reduction of Cr(vi) onto the photocatalysts BW_0_, BW_2,_ and BW_4_ hybrid nanosheets under visible light irradiation

Sample	Malachite Green (MG)				Chromium ion (Cr(vi))			
	First order		Second order		First order		Second order	
	*K* (min^−1^)	*R* ^2^	*k*′ (g (mg^−1^ min^−1^))	*R* ^2^	*K* (min^−1^)	*R* ^2^	*k*′ (g (mg^−1^ min^−1^))	*R* ^2^
BW_0_	−0.016	0.943	1.05	0.955	−0.002	0.188	68.02	0.764
BW_2_	−0.024	0.924	1.33	0.999	−0.006	0.885	8.56	0.978
BW_4_	−0.02	0.908	1.05	0.955	−0.006	0.880	9.84	0.972

**Fig. 11:**
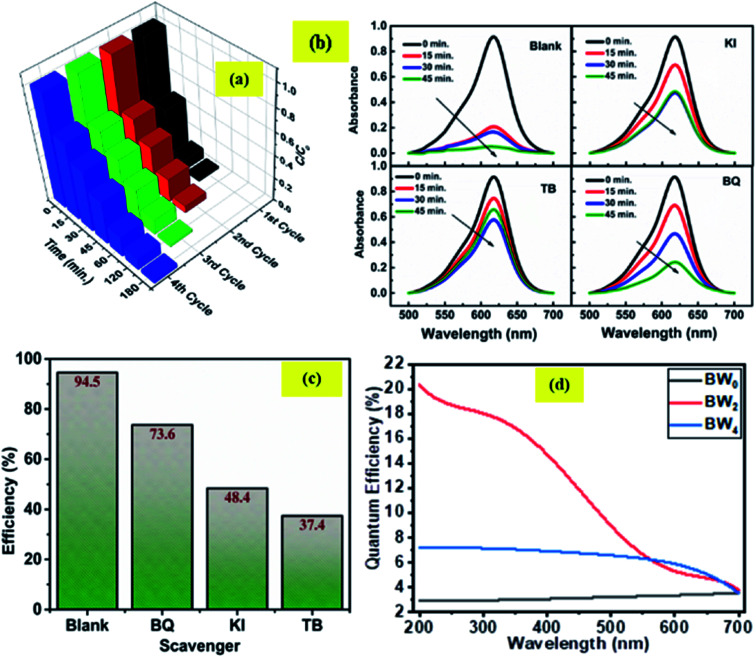
(a) Cyclic tests of photodegradation of MG on BW_2_ hybrid nanosheets. (b) Absorption spectra of MG dye with BW_2_ photocatalyst in the presence of various scavengers (BQ, TB, and KI). (c) Effect of various scavengers in the photodegradation process of MG over BW_2_ nanosheet hybrid and (d) IPCE spectra of BW_0_, BW_2_, and BW_4_ hybrid nanosheets recorded at 0 V *vs.* Ag/AgCl in the wavelength range 200–700 nm.

Four consecutive tests of photodegradation of MG on BW_2_ were run to check the stability of the BW_2_ sample. Fig. 11(a) revealed only 10–20% decrease in photocatalytic performance over the consecutive runs. Hence the BW_2_ exhibit satisfying chemical stability.

### 3.5 Active species trapping experiment

Generally, the photo-induced charge carriers *i.e.* electrons and holes are involved in the photocatalytic process. They get involved in the reaction with H_2_O/HO^−^ and O_2_ and form the superoxide radicals ·OH and ·O_2_^−^ as a product. These active species are fundamentally important in the photocatalytic reactions. In the intention of investigating the active species involved in the photodegradation process of MG using BW_*X*_ hybrid nanosheets, a series of experiments were conducted for trapping the active species in the case of BW_2_ nanosheet hybrid. The different scavengers for different active species *viz.* tetra-butanol (TB) for ·OH, 1,4-benzoquinone (BQ) for ·O_2_^−^, and potassium iodide (KI) for both ·OH and h^+^ were used in the trapping experiments.^72^Fig. 11(b and c) shows the effect of TB, BQ, and KI on the percentage of photodegradation of MG. The addition of the BQ in MG solution had a little effect on the *C*/*C*_0_, indicating that ·O_2_^−^ is the secondary species and not the primary active species during the photocatalytic reaction. On the contrary, the addition of the KI and TB used as ·OH and h^+^ scavenger made a considerable impact on the conversion efficiency. The *C*/*C*_0_ for degradation of MG noticeably decreased after the addition of TB and KI. Therefore, it was established that h^+^ and ·OH have a substantial role in MG degradation. These observations propose that photogenerated ·OH is a dominating player in the MG photocatalytic degradation.

### 3.6 Incident photon to converted electron (IPCE) study

The relationship between the photoactivity improvement and the wavelength of the incident light, and the external quantum efficiency of the samples BW_0_, BW_2_, and BW_4_ were investigated by using Incident Photon to Converted Electron (IPCE) study. In this study, the photocurrent density at each incident wavelength was measured. The IPCE value for a given wavelength was calculated by using the equation14
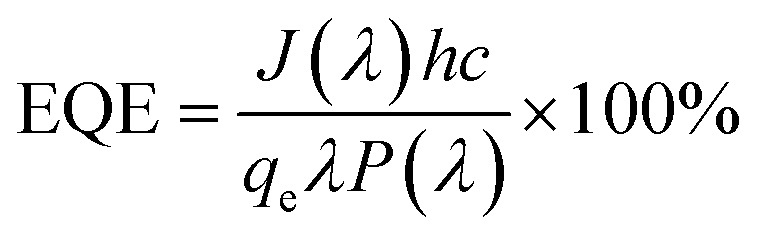
where *J*(*λ*) is the current density as a function of wavelength in A cm^−2^, *h* (*J*s) represents the Planck's constant, *c* (m s^−1^) is the velocity of light. *q*_e_ represents the charge of the electron in *C*, *λ* (nm) represents the wavelength of incident light and *P*(*λ*) (W cm^−2^) represents the intensity of the power that depends on the wavelength of the incident light. Fig. 11(d) displays the IPCE spectra for BW_0_, BW_2_, and BW_4_ hybrid nanosheets. The IPCE values of follows the order: BW_0_ < BW_4_ < BW_2_, in the complete UV-visible spectrum. In particular, the IPCE values of the BW_2_ sample at the shorter wavelength region (<400 nm) were 10 times higher (18–20%) than the IPCE values of BW_0_ nanosheets. This established that the BW_2_ hybrid nanosheet converted the high energy incident photons to electrons well. It is well known that light-harvesting efficiency, charge separation, and collection yields^73^ play an important role in determining the IPCE value of a sample. Thus, we can further infer that the BW_2_ hybrid nanosheets had a larger light-harvesting efficiency and improved charge separation, which was in good agreement with its lower bandgap and slow decay time and better photocatalytic degradation results.

### 3.7 Photocatalytic mechanism

**Fig. 12:**
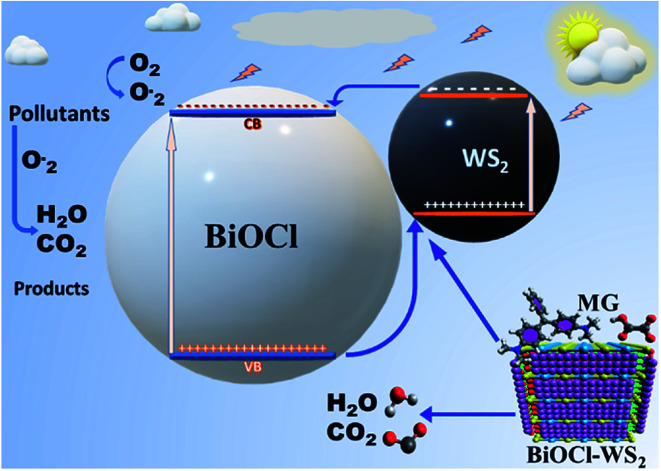
Schematic diagram of photocatalysis mechanism.

After being modified with WS_2_ nanosheets, the BiOCl/WS_2_ nanocomposite showed the higher optical absorption strength in the UV-visible range and larger dye absorption as compared to pure BiOCl nanosheets. The bandgap of BW_*X*_ hybrid nanosheets was also found to be less than the bandgap of pure BiOCl nanosheets. The smaller bandgap of BW_*X*_ hybrid nanosheets is responsible for higher visible light absorption. Due to the higher optical absorption of the BW_*X*_ hybrid nanosheets, the surface adsorption of the dye was also improved. *E*_VB_ and *E*_CB_ corresponding to pure WS_2_ nanosheets are about 2.155 and 0.165 eV, respectively. And for pure BiOCl nanosheets, 3.525 and 0.195 eV, respectively. *E*_CB_ and *E*_VB_ corresponding to WS_2_ are at a higher position than the *E*_CB_ and *E*_VB_ corresponding to pure BiOCl, respectively. So, the electrons that are reaching in the conduction band of WS_2_ can easily transfer to the conduction band of BiOCl and the holes can easily move from BiOCl to the WS_2_ nanosheets (Fig. 12). As depicted by the scavenger test, the photogenerated ·OH radicals play the lead role in the MG photodegradation. The photocatalytic reactions that take place on the surface of BW_*X*_ hybrid nanosheets can be described as follows:BW_*X*_ + *hν* → BW(*X*) (h_vb_^+^ + e_cb_^−^)O_2_ + BW_*X*_ (e_cb_^−^) → ·O_2_^−^ + BW_*X*_2h^+^ + ·O_2_^−^ → 2HO˙2HO˙ → O_2_ + H_2_OMG + h_vb_^+^ + ·OH → H_2_O + CO_2_ + other products.

Photocatalytic activity is boosted with the increase in the charge carrier separation time, resulting in the hindrance to the charge carrier recombination. The time of the separation of charge carriers decides the number of formation of radicals that take part in the photodegradation of the pollutants.

## 4. Conclusion

In conclusion, a low-cost sonochemical route has been applied to synthesize a series of 2D/2D hybrid nanosheets of BiOCl/WS_2_ having high visible-light absorption. The preparation process was simplified efficiently along with reducing the preparation time. The successful fabrication of 2D/2D hybrid nanosheets of BiOCl/WS_2_ is confirmed through the various characterization techniques like XRD, Raman spectroscopy, FTIR spectroscopy, FESEM and EDX. The optical studies including band gap measurement, TCSPC and IPCE confirmed the higher visible light absorption and reduced decay rate of the charge carrier that causes the higher charge transfer rate in the hybrid nanosheets. The as synthesized BW_*X*_ hybrid nanosheets exhibited an enhanced photocatalytic response than pure BiOCl nanosheets for MG photodegradation and Cr(vi) ion photoreduction. The BW_2_ sample achieved the highest photodegradation rate of more than 99% for MG photodegradation in 60 min under visible light irradiation. The major contributing factors in the enhancement of the photocatalytic performance of BW_*X*_ are higher absorption of the visible range, higher rate of charge transfer, larger separation time of photogenerated charge carriers, and high IPCE values in the whole UV-visible spectrum. The photogenerated holes on WS_2_ nanosheets that form ·OH radicals, are found to be the primary active species in the photocatalytic process. The combination of high recyclability, high photocatalytic responsivity in visible light, and non-toxicity of the as-synthesized 2D/2D BW_*X*_ hybrid nanosheet material pave the way for its usage in photocatalysis, and energy renovation applications. This study provides the scope of incorporation of 2D transition metal dichalcogenides in 2D BiOCl nanosheets to assist bandgap engineering and improve the rate of charge transfer for better photocatalytic activity.

## Conflicts of interest

There are no conflicts to declare.

## Supplementary Material
